# Simulated Comparison of On-Chip Terahertz Filters for Sub-Wavelength Dielectric Sensing

**DOI:** 10.3390/s26010129

**Published:** 2025-12-24

**Authors:** Josh Paul Robert Nixon, Connor Devyn William Mosley, Sae June Park, Christopher David Wood, John Cunningham

**Affiliations:** 1 School of Electronic and Electrical Engineering, University of Leeds, Leeds LS2 9JT, UK; 2 School of Electronic Engineering and Computer Science, Queen Mary University of London, London E1 4NS, UK

**Keywords:** terahertz sensors, Ansys HFSS, THz-TDS, split-ring resonators, terahertz imaging

## Abstract

This paper discusses the application of on-chip terahertz (THz) filters attached to waveguides that can act as sensor elements, including for scanned imaging applications. Our work presents a comparative numerical study of several different geometries (comprising five split-ring resonator geometries and a quarter-wavelength stub resonator, the latter being well established as a sensor at THz frequencies and therefore able to act as a benchmark). We designed each structure to have a resonant frequency of 500 GHz, allowing the impact of resonator geometry on sensing performance to be isolated; the performance was quantified by assessing each design using four figures of merit: resonance quality factor, sensitivity (relative frequency shift under dielectric loading), responsivity (sensitivity weighted by resonance sharpness), and the electric field confinement area. Simulations were conducted using Ansys HFSS using the properties of a commercially available photoresist (Shipley 1813) as a dielectric load to assess performance under conditions comparable to previous experimental studies. The analysis showed that while sensitivity remained broadly similar across geometries, responsivity and quality factor differed substantially between resonators. Furthermore, the spatial distribution of the electric field and current density, particularly in rotated configurations, was found to significantly impact coupling efficiency between the resonator and transmission line. Our findings provide guidance for the general design of systems employing THz sensors while establishing a framework with which to benchmark future sensor geometries.

## 1. Introduction

Terahertz (THz) sensing and imaging techniques have emerged as powerful tools for the non-invasive characterisation of materials [1] including semiconductors [2], drugs, explosives [3], polycrystalline materials [4], and biological materials [5]. In the biomedical domain, THz radiation is particularly attractive for imaging and sensing, because its low photon energy avoids ionising tissue damage, while its sensitivity to water content and molecular vibrations enables label-free identification of diseased tissues as well as the capability to distinguish between different biomolecules. Applications of this explored to date include detection of skin cancer [6,7], monitoring of burn healing [8], and assessment of hydration in tissues [9]. These capabilities position THz technologies as a promising platform for medical diagnostics, disease monitoring, and in vivo biological analysis.

The experimental techniques commonly used in the THz domain can present challenges in certain applications, including in the measurement of small-volume analytes or of samples located in difficult-to-access spaces, such as in vivo sensing [10]. In THz time-domain spectroscopy (THz-TDS), for example, free-space optics such as lenses or parabolic mirrors are typically used to direct THz radiation from a source to a sample and on to a detector. The required optics for the THz beam are physically bulky, and the spatial resolution of measurements is constrained by the diffraction limit (typically 100 s of μm) without resorting to near-field probe techniques [11,12]. Furthermore, free-space THz-TDS measurements require a controlled, low-humidity environment owing to the high absorption of THz radiation by atmospheric water vapour [13]. However, all these constraints can be mitigated by using on-chip THz-TDS technology, in which an evanescent electric field accompanies a picosecond-duration current pulse that propagates within a waveguide, thereby eliminating the need for free-space THz optics [14,15]. On-chip THz-TDS has been applied to the measurement of small material volumes (<0.1 μm^3^) [4,16] and of non-uniform materials, with a spatial resolution well below the diffraction limit [17], and defined by lithographic considerations for the geometry of the chosen waveguide [18]. In addition, we recently demonstrated that asynchronous optical sampling allows video-rate measurement of on-chip THz time-domain signals [19], offering a route to real-time sensing and imaging applications using these same techniques.

**Figure 1:**
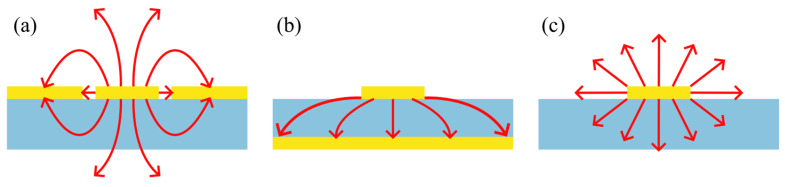
Schematic diagrams of the most common instantaneous electric field modal patterns for the waveguides discussed in the text: (**a**): CPW; (b): MSL; (**c**): PGL. Red arrows show electric field, while blue regions are dielectric substrate, and yellow regions are conductors.

Several geometries for on-chip THz waveguides have been reported, including coplanar waveguides (CPWs) [4], microstrip lines (MSLs) [20], and planar Goubau lines (PGLs) [21], each seen with their respective electric field modes in Figure 1. In addition to metal-based waveguides, silicon-based THz waveguides and resonant cavities have recently demonstrated strong potential for integrated sensing and filtering, with topological cavity designs achieving robust and high-Q resonances [22]. Each type of waveguide supports different transmission modes which localise the coupled evanescent field in different ways. In both CPW and microstrip devices, for example, the propagating electric field is confined between adjacent conductors, comprising a signal transmission line and one or more ground planes. In a CPW, the majority of the electric field is constrained between a central conductor and parallel, in-plane ground conductors, whereas in MSLs, the field is confined primarily within the substrate, between a transmission line on the top surface and a ground plane on the rear. In silicon waveguides, this localisation is dominated by strong dielectric confinement, with most of the THz mode contained within the high-index core and only a relatively small evanescent field accessible at the surface. In contrast, PGLs are single-conductor waveguides that support a Goubau mode, characterised by an electric field that extends radially from the transmission line, and a magnetic field which circulates around the PGL. This makes the PGL system a particularly attractive prospect for THz sensing applications, as the evanescent field extends further above the device, which may induce a stronger interaction with any overlaid material. The integration of resonant structures into the THz waveguide enables frequency-selective filtering of transmitted pulses [23], with a resonant frequency that is highly sensitive to the local dielectric environment. Such resonant structures can therefore function as sensors and then be used to measure the presence of analytes or to map changes in material surface composition. Resonators also further confine the evanescent field, improving the effective spatial resolution of the sensor compared to a bare waveguide. However, their high field confinement may also limit interactions between the THz electric field and an overlaid analyte, and therefore, careful selection of both the resonator and waveguide geometries is required.

The two types of resonators discussed in this paper are split-ring resonators (SRRs) and quarter-wavelength stub (QWS) filters. SRRs can be understood using a simple lumped-element model: The metallic ring forms an inductive loop, while the split or capacitive plates generate a capacitance across the gap. Together, these elements behave as an effective LC circuit with a resonance frequency $f_{0}=1/2\pi \sqrt{LC\varepsilon_{eff}}$, where inductance L is set primarily by the ring geometry and enclosed magnetic flux, and capacitance C is governed by the gap width, plate area, and local dielectric environment. QWSs, by contrast, work by inverting the phase of incident propagating waves. At resonance, this phase inversion interferes destructively with specific wavelength components, attenuating the corresponding frequency. The resonant frequency is given by $f_{0}=c/4l\sqrt{\varepsilon_{eff}}\;$ and therefore depends on the stub length l and the effective permittivity of the surrounding medium, $\varepsilon_{eff}$. Since the resonance conditions of both SRRs and QWSs depend on the dielectric properties of materials in their vicinity, measured shifts in their resonant frequencies relative to a reference dielectric can be used directly as a sensing mechanism.

To our knowledge, no detailed study of THz resonator architectures, suitable for use with PGL waveguides, is available. Previous studies of resonant THz sensors have instead focused on optimising a single resonator geometry for a specific application [24,25,26]. This likely stems from the complex, interdependent parameter space governing resonator geometry and performance. All design parameters which dictate the inductance and capacitance of the resonator also influence the fundamental resonant frequency of the structure and the distribution of the electric and magnetic fields. However, the fundamental frequency directly affects the sensitivity of the resonator to dielectric loading and therefore cannot be decoupled from the basic design. To reduce the parameter space and allow a fair comparison, all resonators presented here were designed to operate with the same resonance frequency of 500 GHz. Fixing the resonance frequency then enables a quantitative evaluation of factors such as sensitivity, responsivity, electric field confinement, and tolerance to performance degradation in lossy dielectrics. Although this study focuses on this 500 GHz resonance, the underlying physical mechanisms examined are not specific to the frequency. Relative behaviour of the resonator geometries is governed primarily by their geometry and by the evanescent-field interactions, rather than by the absolute operating frequency. We therefore expect the trends identified in this work to extend across the broader THz range, provided the resonators are appropriately scaled. In addition, multi-band sensing using cascaded resonators operating at different frequencies along a single PGL has previously been demonstrated [23], and the design principles presented here readily apply to such multi-resonant configurations.

This frequency-controlled comparison will provide valuable design guidance for future THz sensors while establishing a reproducible framework with which to benchmark future resonator-based devices.

## 2. Methods

**Figure 2:**
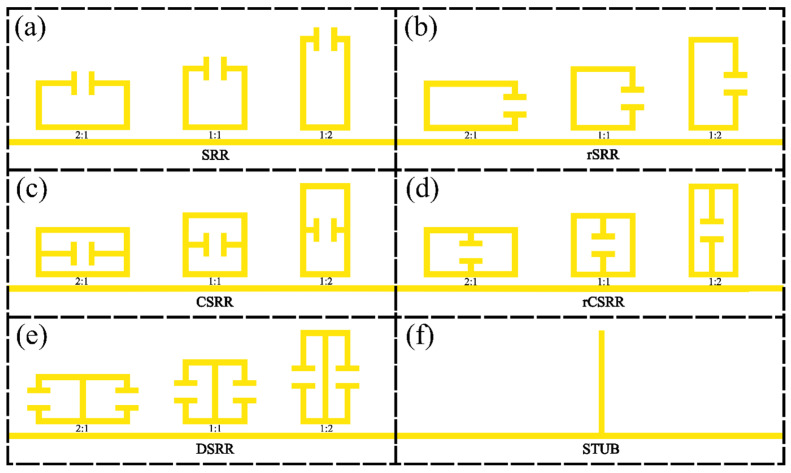
Schematic diagrams of all the resonator types simulated in this work, and their orientation relative to the Goubau line. (**a**–**f**): Each panel shows the three aspect ratios chosen for the SRRs shown. The size of each resonator is defined in the text.

**Figure 3:**
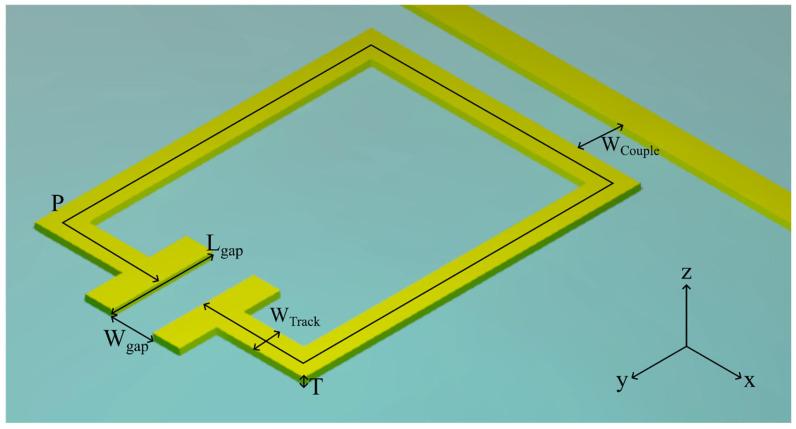
Diagram of split-ring resonator design with parameters and coordinate system labelled.

Figure 2 shows a schematic diagram of all resonator geometries investigated in this work, which were chosen owing to their commonality in the literature, both for the THz and microwave frequency ranges [27,28,29]. While other geometries are found in the literature, this work provides a general methodology for comparison, and the 6 chosen aim to show that despite the differences in overall structure, rather than differences in certain parameters, the methodology allows fair comparison. The geometries are as follows and shown in Figure 2: (a) split-ring resonators (SRRs); (b) rotated split-ring resonators (rSRRs), in which the capacitive gap is oriented at 90° relative to the SRRs in (a); (c) centre-split-ring resonators (CSRRs); (d) rotated centre-split-ring resonators (rCSRRs), where the capacitive gap is oriented at 90° relative to the CSRRs in (c); (e) double-split-ring resonators (DSRRs); and (f) the QWS resonator. As also shown in Figure 2, we can further categorise each SRR by its aspect ratio *AR* = *w*:*h*, where width is defined parallel to the transmission line, for variations AR = 2:1, 1:1, and 1:2. Exploration of these variations provides a better understanding of how the capacitive gap orientation and inductive perimeter affect resonator performance and field distribution. The QWS resonator was included as a benchmark design commonly found in the literature. It has a much smaller design-parameter space that can be readily optimised for a specific frequency, and its inclusion therefore also allows for comparison between LC resonances and its standing-wave resonance. The dimensions of the QWS are given by its length (93.2 μm), width (5 μm), and thickness (110 nm), chosen to also achieve an unloaded resonance at 500 GHz. For all split-ring resonators studied, certain dimensions (seen in Figure 3) were fixed for comparison, including the resonator conductor width *W_Track_* = 2 μm, metal thickness *T* = 110 nm, capacitive arm length *L_gap_* = 20 μm, capacitive gap width *W_gap_* = 6 μm, and coupling gap width *W_Couple_ =* 1.5 μm. The PGL transmission line (also pictured in Figure 3) extended the full length of the simulation domain, and had a width of 5 μm and the same metal thickness T as the resonators. The inductive perimeter *P* is given by *P* = *aP*_0_, where *P*_0_ is the initial inductive perimeter (*P*_0_ = 100 μm), while *a* is a scaling factor used (0.25<a<1.25). The *W_Couple_* parameter specifically is one which could affect resonator performance. It was chosen to be 1.5 μm, since this is both a small enough gap to ensure reasonably high coupling efficiency, and a large enough coupling gap size to ensure that these resonators are manufacturable under standard lithographic conditions. We note that trends observed when comparing resonator geometries should remain consistent for any *W_Couple_* value, so long as this value is maintained between different resonator geometries.

Finite element simulations of each resonator geometry were carried out using the Ansys High Frequency Structure Simulator (HFSS). The simulation volume was a cuboid (500 μm × 500 μm × 300 μm) which fully contained the on-chip waveguide, filters, and wave ports used to define the signal excitation and extraction wavevectors. The simulation used a quartz substrate with HFSS’s default property values (ε_eff_ = 3.78, tan(δ) = 10^−5^), with a thickness of 200 μm. Two-port S-parameter simulations were used to obtain S_21_ for each resonator. Wave ports were directly coupled to the start and end of the PGL, with the electric field defined as pointing radially away from the PGL to induce a Goubau mode [30]. S_21_ acted as a measure of how much signal was received at the second port (the output) relative to what was inputted to the device at the first port. Radiation boundaries were used to suppress reflections from the simulation edges, ensuring accurate field propagation. The simulation spatial mesh was generated using HFSS’s adaptive convergence algorithm; the RMS edge length for mesh elements ranged between 0.5 and 0.8 μm for all resonator geometries where the field complexity was the highest. Numerical stability was ensured using HFSS’s adaptive meshing algorithm. Specifically, adaptive passes continued until the change in the S-parameters between successive meshes satisfied the HFSS convergence criterion (ΔS_21_ < 0.005 across the full frequency band). At this point, further mesh refinement did not produce any appreciable change in the extracted resonance frequency or Q-factor. Each resonator was then evaluated using the following procedure: Starting with a 1:1 aspect ratio, the inductive perimeter length (*P* in Figure 3) was scaled by multiplying the initialisation perimeter *P*_0_ by a scaling factor *a* while maintaining the aspect ratio and all other constrained dimensions. The resonant frequency versus scaling factor was then fitted using a linear regression in the range 480–520 GHz, allowing the scaling factor *a* required to achieve a 500 GHz resonant frequency to be determined for each design. The residual standard error of this linear regression was *RSE*(*a*) < 0.01 across all resonator geometries and aspect ratios, and the interpolated value for *a* was found to give a resonant frequency of 500 GHz ± 1 GHz. This procedure was then repeated for each aspect ratio. Dielectric loading of each filter was next investigated by adding a layer of the Shipley 1813 photoresist (S1813) [31] on top of the device, spanning the simulation space in a uniform square 500 × 500 μm, with a thickness varying from 0 (representing an unloaded filter) to 100 μm (by which thickness the evanescent field is fully enclosed in all cases). S1813 was chosen for its prior use in determining the sensitivity of on-chip THz sensors [23], allowing a direct comparison with our simulations. The dielectric properties (permittivity and loss tangent) of S1813 used were ε = 2.7 and tan(δ) = 1.5 at 500 GHz, as determined previously by an experiment [32]. The relatively high loss tangent of S1813 also allows us to test the relative reduction in Q-factor of the resonant response of each geometry to dielectric loss, which low-loss materials would not provide. Furthermore, it has been shown in previous studies [33] that trends observed should be replicable for other values of the refractive index, so that calculations of the FOMs hold well for other materials. For each resonator, the transmission parameter S_21_ between 430 and 510 GHz and the electric field distribution on resonance were determined for comparative analysis. It should be noted that in the case of non-uniform coverage of the sensor, the frequency shift of the resonator would be reduced as dielectric interactions with the sensor are reduced. Hence, any experiment intending to reproduce these results would require uniform film deposition, and care taken to remove any air gaps between the dielectric and sensing element.

**Figure 4:**
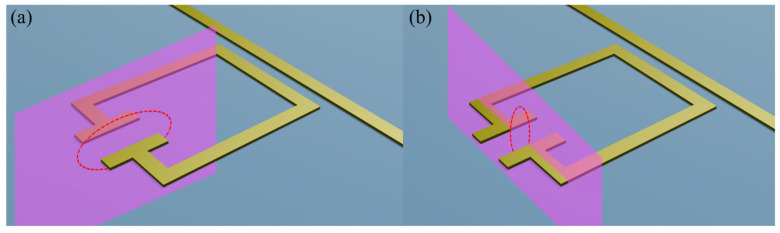
Schematic diagram showing the planes in which the electric field distribution was extracted, overlaid on an example resonator for clarity. Panel (**a**) depicts the y-z plane, while panel (**b**) depicts the x-z plane.

We define the FOMs used to assess sensing performance as follows: Q-factor—$Q=f_{0}/FWHM$, where $f_{0}$ is the resonant frequency and FWHM is the full width at half maximum of the resonance; sensitivity—S=∆f/∆n, where ∆f is the shift in the resonant frequency of the device under dielectric load and ∆n is the change in refractive index (here ∆n=0.62 from air to S1813); and responsivity—R=S/FWHM, where FWHM is the full width at half maximum of the filter absorption in the loaded case. Sensitivity and responsivity quantify the frequency shift and absorption change as a function of refractive index variation [34], respectively. We define effective sensing volume *V* of our filters by the localisation of the evanescent field around the resonator. This we quantify by evaluating the spatial extent of the on-resonance evanescent electric field surrounding the resonator. A colourmap of the electric field magnitude was generated in the y-z and x-z planes (centred in the middle of the capacitive gap), defined as parallel and perpendicular to the capacitive plates, respectively (Figure 4). An elliptical boundary was defined on each plane, which defined the area within which the field magnitude remained greater than 1/*e* of its maximum value [10], shown in Figure 4. These two areas were then used to define the effective sensing volume of the evanescent field, given by $V=\sqrt{A_{yz}*A_{xz}}$, where *A_yz_* and *A_xz_* are the areas of each ellipse. The effective sensing volume allows us to quantify the spatial resolution of each resonator geometry. Finally, we also define a combined CFOM as CFOM=ΔQS/V to aid easy quantitative comparison of the overall sensing performance. The addition of the CFOM provides insight into overall trends, and aids in choosing a practical generalised sensing-element geometry. However, comparison of each individual FOM allows us to instead suggest best-use cases for each resonator type, as we discuss later.

## 3. Results

**Figure 5:**
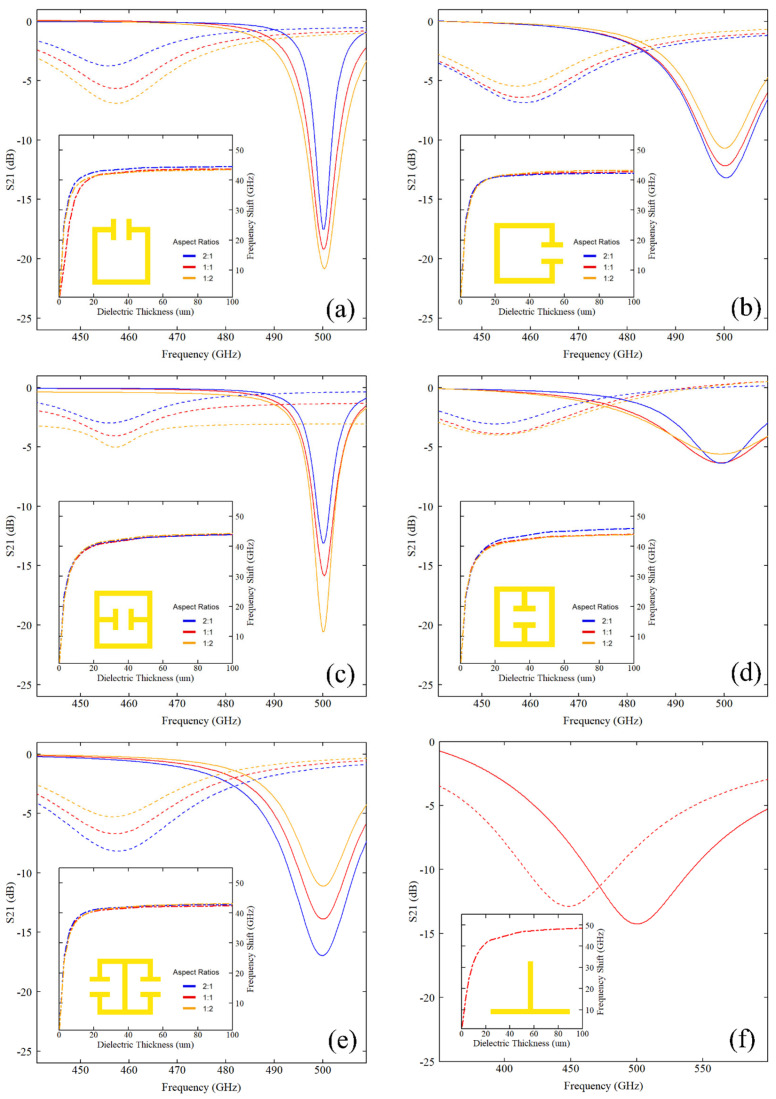
S_21_ transmission parameter plots for all explored resonator shapes and aspect ratios ((**a**): SRR; (**b**): rSRR; (**c**): CSRR; (**d**): rCSRR; (**e**): DSRR; and (**f**): stub). Solid lines show the unloaded resonator, while dashed lines show the response with 100 μm of overlaid Shipley 1813. Insets show resonant frequency shift of each geometry as a function of dielectric thickness. Curves were fitted using non-linear least-squares regression. The fit returned the resonance frequency $f_{0}$ and full width at half-maximum (FWHM), together with their standard errors. The FOMs were then calculated with standard error propagation using the uncertainties on $f_{0}$ and FWHM, and the largest uncertainty is seen at the top of each column.

The forward transmission parameter S_21_ for each resonator is presented in Figure 5, which includes S_21_ for each unloaded resonator (solid lines) and for a 100 μm thick overlaid layer of S1813 (dashed lines). For each case, the loaded resonance frequency is down-shifted by 40–50 GHz from resonance upon loading with S1813. Insets in Figure 5 show the corresponding evolution of this frequency shift as the dielectric thickness is increased from 0 μm to 100 μm for each different aspect ratio. In each case, the frequency shift is observed to change rapidly for thin layers of the dielectric, before saturating as dielectric thickness increases. Plots are labelled (a)–(f) in accordance with the Figure 2 labels. The data from Figure 5 were used to calculate the FOMs Q-factor, sensitivity, responsivity, and sensing volume for each resonator geometry, which are summarised in Table 1.

## 4. Discussion

**Figure 6:**
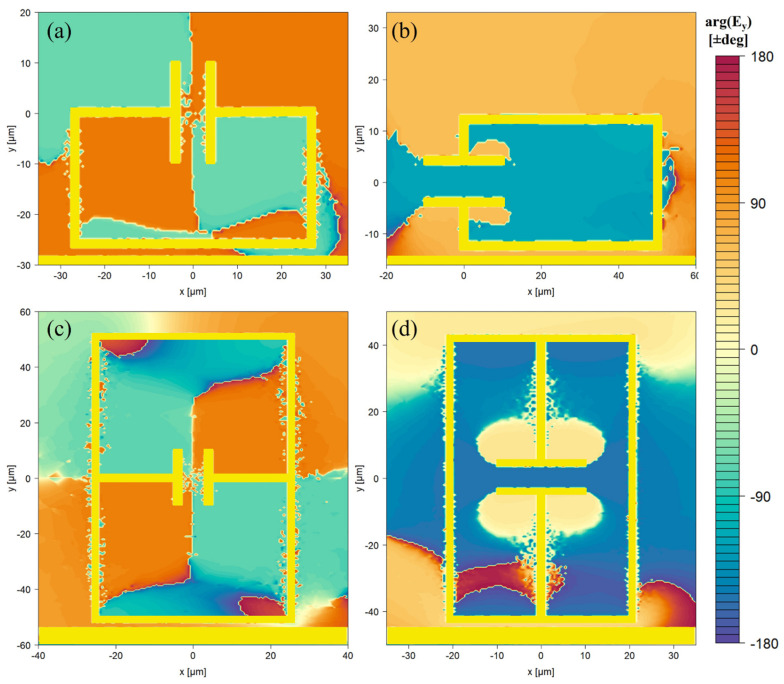
Phase distribution of the y-polarised portion of the electric field for the 2:1 (**a**) SRR and (**b**) rSRR, and the 1:2 (**c**) CSRR and (**d**) rCSRR.

Each of the LC-resonance structures exhibits an unloaded Q-factor ranging between 18 and 130, with the highest Q-factor occurring in the 1:2 variant of the SRR and CSRR, whilst the lowest occurs in the 1:2 variant of the rCSRR. Upon loading with 100 μm of S1813, the Q-factor of all resonators is observed to decrease, as expected for interaction with a lossy dielectric. This can be observed in the reduction in absorption magnitude and the broadening of the resonances seen in Figure 5a. Greater relative reduction in Q-factor was found for the structures that have a higher unloaded Q-factor, as seen in the ΔQ-factor column. In comparison to the LC-resonance structures, the QWS produced a lower unloaded Q-factor of 5.5, though we note that its ΔQ-factor is low compared to the split-ring designs; i.e., the Q-factor of the stub appears to be less affected by interaction with the lossy dielectric. A clear trend can be observed whereby LC resonators with the capacitive gap oriented parallel to the transmission line (SRR and CSRR) produce a higher Q-factor compared to the designs which have the capacitive gap oriented perpendicular to the transmission line (rSRR, DSRR, rCSRR). In addition, designs in which the capacitive gap is located parallel to the PGL (1:2 aspect ratio) produce higher Q-factors for almost all structures. The variation in response observed upon rotation is related to the polarisation of the E-field between the transmission line and the resonator. Figure 6 maps the phase of the electric field component aligned with the transmission line, arg(Ey), at resonance.

The Goubau-line fundamental mode exhibits E-fields normal to the conductor surface (as seen in Figure 1c), with H-fields encircling it. When the capacitive plates are oriented perpendicular to the transmission line (Figure 6a,c), the resonator interior splits into two phase domains of opposite sign. This antisymmetric field establishes a large voltage across the capacitive gap, excites a circulating current (LC),and concentrating magnetic flux through the loop, which promotes inductive coupling between the transmission line and the resonator, and suppresses the net radiating dipole, thereby increasing Q. In contrast, with plates parallel to the transmission line (Figure 6b,d), the E-field inside the resonator is almost purely single-phase: charges move in phase along the metal, and the capacitive gap potential difference is reduced, so that the coupling between the transmission line and resonator is dominated by capacitive coupling aligned with the electric field from the transmission line. The resulting increase in dielectric interaction leads to lower Q [35,36]. These observations lead us to propose a consistent design rule: capacitive sections should be orthogonal to the transmission-line E-field to maximise energy confinement in the presence of dielectric loading.

A consistent sensitivity of 68–73 GHz/RIU was observed across all LC-resonator geometries, whilst the stub filter exhibits a slightly higher sensitivity of 85 GHz/RIU. The SRR and CSRR structures produce the highest responsivity values of the LC-resonance structures at 2.7–3.3, whilst the rSRR and rCSRR produce the lowest value of 1.9. This correlates with the trend in the Q-factor discussed above, in that the capacitive gap being oriented parallel to the transmission line produces sensors with higher responsivity. By contrast, there was no clear trend in the responsivity when the aspect ratio was varied between the different structures. The QWS showed a responsivity of 0.6, however, lower than all the SRRs. The sensing volume for the SRRs varied between 107 and 120 μm^3^, with no clear trend with respect to either the capacitive gap orientation or the aspect ratio of the structures. In comparison, the QWS achieves the smallest sensing volume of all the structures investigated at 6.3 μm^3^. However, we note that despite the region of the high field being localised at the QWS tip, the entire length of the QWS is sensitive to dielectric changes. More detailed simulations are needed to fully quantify the impact of the most sensitive region at the tip in comparison to the larger, less sensitive region along the QWS. The overall behaviour of the QWS can be understood by considering the standing-wave field distribution along it. The antinode of the standing wave is found at the tip of the QWS, thereby producing the highly localised region of a strong evanescent field as observed. The concentration of the evanescent field allows for strong penetration of the field into the overlaid dielectric, resulting in a larger change in effective refractive index when the layer is introduced, and hence a higher sensitivity. However, this higher penetration comes at the cost of energy confinement and therefore Q-factor. The QWS trades resonance sharpness for stronger field–environment coupling, which manifests as higher sensitivity but lower Q-factor.

Across all LC-resonator geometries in this study, the 1:2-aspect-ratio SRR and CSRR are the resonators most suited to applications requiring high selectivity in the dielectric properties of materials under test, owing to their high Q-factors and responsivities. In comparison, the QWS exhibited lower responsivity and Q-factor relative to the other simulated sensors, meaning it is less suited for applications requiring precise frequency selectivity. Instead, its utility is clearly in applications where material differentiation with high localisation is needed, since it has the lowest effective sensing volume. This is especially true for sensing of very lossy overlaid materials, where the split-ring designs showed greater reductions in absorption magnitude and Q-factor when compared to the stub.

To validate the accuracy and experimental relevance of the simulated resonator responses, we compare our results to previous numerical and experimental studies on THz resonators and filters. The simulated quality factors, sensitivities, and field confinement characteristics align closely with values reported in prior numerical investigations of split-ring resonator designs operating at comparable frequencies. Specifically, Ref. [10] shows that similar electric field confinement along the z-axis was observed for a 2:1 rectangular split-ring resonator (rSRR) with a capacitive gap width of 6 μm (the effective sensing height being 4.5 μm in [10] and 4.7 μm in this paper, with the value extracted to calculate the sensing volume seen in Table 1 for the 2:1 SRR), with only a ~5% difference when accounting for the differing loss tangents of the protective dielectric layers used therein (Parylene-C versus polyimide). The remaining discrepancy can be attributed to small variations in geometric parameters, such as metallisation thickness and inductive perimeter length. After accounting for differences in the permittivity of the dielectric loading materials (Shipley 1813 in this work versus colon tissue in Ref. [10]), the simulated sensitivities for equivalent resonator geometries are also in good agreement, differing by less than 10% (between 68 and 73 GHz/RIU for this paper, and ~64 GHz/RIU for Ref. [10]). This level of agreement is consistent with previously reported experimental variability in resonator-based sensing platforms. Experimentally, resonators for sensing applications are more commonly investigated in the microwave regime [37,38]; however, unloaded Q-factors ranging from 36 to 167 have been reported for THz split-ring resonators excited via microstrip feeding or free-space coupling [25,39], which is consistent with the unloaded and loaded Q-factors obtained in the present simulations.

Notably, in Ref. [25], Shipley 1813 was used to load a resonator closely related to the rCSRR studied here. That work reported a resonant frequency shift of approximately 40 GHz, corresponding to a sensitivity of ~70 GHz/RIU, in excellent agreement with the simulated values presented in Table 1 (between 68 and 73 GHz/RIU for split-ring designs) of this paper. Furthermore, experimental investigations of a stub resonator in Ref. [29], while utilising a microstrip excitation scheme, demonstrated very similar FWHM values (~90 GHz in [29], compared with 91.5 GHz in these simulations).

The sensor designs simulated in this work may be readily fabricated using standard nanofabrication techniques. For example, the fabrication of an on-chip PGL device with a QWS resonator was reported in reference [40], and this process could be adapted for the SRRs presented in this work. Experimental verification of the simulated S_21_ response of a THz microstrip device has previously been reported in reference [29], using THz-TDS. Device designs which integrate photoconductive switches into the THz waveguide, such as those reported in references [29,40], permit measurement of the THz pulse at both the input and output of the waveguide, from which S_21_ may be calculated. The experimentally measured S_21_ in Ref. [29] was found to be in good agreement with simulations. Such experimental methods therefore present a straightforward route to experimentally verifying the simulations presented in the current work.

## 5. Conclusions

We have investigated the response of several common resonator geometries for their use as THz sensors when integrated with a PGL transmission line. Specifically, and in contrast to previous studies, we compare different resonator geometries which all have an unloaded resonance of 500 GHz by design, allowing us to isolate the effect of geometry on sensing performance. The relative orientation between the capacitive gap and the PGL was found to play a critical role in resonator efficiency, with orientations placing the capacitive gap parallel to the transmission line consistently producing higher Q-factors and responsivities. We also compared the performance of SRRs with QWSs, demonstrating that whilst the former LC-resonance structures produce sensors with generally higher quality factor and sensitivity, the QWS was found to be preferred for applications needing high spatial resolution. Our results are found to be consistent with previous studies of on-chip THz sensors. Overall, our comparative study provides a framework for selecting resonator geometries based on their intended sensing application. Future work could explore these geometries under spatially heterogeneous dielectric environments to further assess their real-world applicability, which is expected to be important for applications including the imaging of complex systems such as biological tissues.

## Figures and Tables

**Table 1:** Figures of merit obtained for all resonator shapes and aspect ratios in the order (1–6) displayed in Figure 2 and Figure 5 columns given by previously stated formulae (from left to right): AR = w/h $Q=f_{0}⁄FWHM$; ΔQ = Qunloaded − Qloaded; S=∆f⁄∆n; R=S⁄FWHM; $V\;=\;\sqrt{A\_yz*A\_xz}$; and *CFOM =* ΔQ∗S/V.

Resonator Geometry	Aspect Ratio	Q-Factor (Loaded/Unloaded) (±0.4)	ΔQ (±0.6)	Sensitivity (GHz/RIU) (±0.2)	Responsivity (±0.03)	Sensing Volume (µm^3^)	CFOM (±0.4)
**1. Split-Ring Resonator**	2:1	18/67	49	70	2.7	109	31
	1:1	19/77	58	70	2.9	115	35
	1:2	20/130	110	72	3.2	115	69
**2. Rotated Split-Ring Resonator**	2:1	13/29	16	68	1.9	107	10
	1:1	13/29	16	68	2.0	107	10
	1:2	14/31	17	69	2.2	109	11
**3. Centre-Split-Ring Resonator**	2:1	21/110	89	71	3.3	118	54
	1:1	21/97	76	71	3.1	107	50
	1:2	19/130	111	72	3.1	112	71
**4. Rotated Centre-Split-Ring Resonator**	2:1	17/31	14	73	2.7	110	9.3
	1:1	13/21	8	72	2.1	111	5.1
	1:2	12/18	6	72	1.9	108	4.0
**5. Double-Split-Ring Resonator**	2:1	14/36	22	69	2.1	108/120	14/12
	1:1	15/36	21	68	2.2	112/116	13/12
	1:2	16/37	21	69	2.4	107/116	14/12
**6. Stub Resonator**	N/A	3.2/5.5	2.3	85	0.6	6.3	31

## Data Availability

The data associated with this paper are openly available from the University of Leeds Data Repository at doi:10.5518/1790.
